# Integrating Dietitians Into Primary Dental Care for Paediatric Populations: Developing a Conceptual Service Pathway Through a Professional Stakeholder Workshop

**DOI:** 10.1111/jhn.70361

**Published:** 2026-10-09

**Authors:** Lauren Hallewell, Raul Bescos, Zoe Brookes, Robert Witton, Patricia Casas‐Agustench

**Affiliations:** ^1^ School of Health Professions, Faculty of Health University of Plymouth Plymouth UK; ^2^ Peninsula Dental School, Faculty of Health University of Plymouth Plymouth UK

## Abstract

**Background:**

Diet is a common risk factor influencing both oral and systemic health, yet dietary support within dental care is often limited to brief advice focused on sugar intake. Integrating dietitians into primary dental care for the paediatric population may support earlier identification of dietary concerns and provide the opportunity for dietetic care. However, no established integrated dietetic‐dental service pathway for paediatric populations has been identified within primary dental care settings in the United Kingdom.

**Objective:**

To explore how selected full‐consensus agreement statements could be configured into a conceptual dietetic‐dental service pathway for paediatric populations attending primary dental care services and to identify the organisational and contextual factors that may influence its future delivery in practice.

**Methods:**

A single 70‐min online workshop was conducted via video conferencing in April 2026 and facilitated using a structured discussion guide. Workshop activities focused on collaborative service pathway mapping informed by previous Delphi consensus findings. Data sources included a workshop transcript, digital sticky‐note outputs, pathway mapping artefacts, facilitator notes and analysed using framework analysis. Subsequent member checking was undertaken to verify the accuracy and completeness of the developed care pathway.

**Results:**

Eight professional participants (four dietitians and four dental professionals) contributed to the development of a conceptual service pathway. The pathway centred on dietitian‐led triage as a central coordinating mechanism, supporting referral management, communication and continuity across services. Workshop outputs highlighted a range of contextual factors that may influence future delivery, including patient engagement, professional roles and system readiness. Key uncertainties related to workforce configuration, escalation pathways and the capacity of external services to accommodate potential referrals generated through preventive dietary care.

**Conclusion:**

The findings represent an early stage conceptual dietetic‐dental service pathway for paediatric populations, requiring future piloting and evaluation. Contextual factors suggest that delivering the care pathway may require adaptive approaches that are responsive to local workforce organisational and population contexts.

## Summary

- Integrated dietetic care within primary dental services may identify children whose needs extend beyond the capacity of a single dietetic practitioner, requiring links with wider dietetic and multidisciplinary services.
- Workforce configuration, information sharing processes and the capacity of existing dietetic services should be considered and reported during the planning and piloting of integrated dietetic‐dental care models.
- Pathway mapping identified dietitian‐led triage as an important mechanism for coordinating referrals, determining care pathways and supporting continuity of care.

## 1 Introduction

Diet is a modifiable risk factor for dental caries, which remains the most prevalent non‐communicable disease globally. In the United Kingdom (UK), dental extractions are the leading cause for general anaesthesia in children [1]. In paediatric populations, primary dental care settings provide critical touchpoints to support healthy dietary behaviours during developmental stages when lifelong health habits are established. However, dietary advice delivered within dental settings is often narrowly focused on sugar intake and frequency, with limited scope for broader nutritional support [2]. Dietary discussions led by dental teams can be constrained by professional confidence, reimbursement, nutrition training and appointment times, limiting the provision of sustained dietary support beyond brief advice [3]. The success of dietary modifications is strongly influenced by patient socio‐economic factors, caregiver knowledge, and motivation, yet these critical aspects are often overlooked in clinical dental practice [2].

Registered dietitians are trained to assess, diagnose and manage dietary and nutritional problems across the life course [4]. As integral members of multidisciplinary teams (MDT), dietitians utilise evidence‐based approaches to improve nutritional outcomes and support behaviour change, positioning them well to support dietary management within primary dental care [4, 5]. Integrating dietitians through physical co‐location within primary dental care alongside screening for common dietary risk factors within dental settings may enable earlier identification of families at increased risk of both oral and systemic health conditions, facilitating timely access to appropriate healthcare support [6]. Such integration may support more holistic and preventive models of paediatric care by addressing shared risk factors across oral and systemic health outcomes [5]. Research suggests that integrating nutrition expertise within dental settings is generally acceptable to caregivers, particularly when focused on oral health [7]. Similarly, adult dental patients have reported positive perceptions of receiving dietary counselling from nutritionists within dental clinics, valuing personalised dietary advice and ongoing follow‐up support for oral and systemic health [8].

Despite increasing calls for interprofessional collaboration between dietitians and dental professionals, there remains no clearly defined model for integrating dietitians within primary dental care [5, 9]. A published model of paediatric dietetic‐dental integration was described within an American university dental clinic and focused on student training [10]. As such, evidence of an established integrated dietetic‐dental care pathway for paediatric populations within UK primary dental care settings remains limited. Focusing on paediatric populations (0–18 years), where early prevention and family‐centred care are critical, previous qualitative work indicated that integration is acceptable when implemented collaboratively, sensitively and with appropriate structural support [7]. A subsequent Delphi established consensus on key components for integration, including regarding referral criteria and processes, communication pathways, training requirements and structural and organisational considerations [11]. Full consensus agreement was defined as agreement achieved across dietitians, dental professionals and the overall panel [11]. However, it remains unclear how selected full‐consensus agreement statements can be configured into a conceptual service pathway, including the contextual and organisational factors that may influence future delivery in practice. Therefore, this study aimed to explore how these statements could be configured into a conceptual service pathway for paediatric populations attending primary dental care services.

## 2 Methods

### 2.1 Study Design

A single structured online workshop was conducted via video conferencing (Microsoft Teams) in April 2026 to maximise accessibility and provide a neutral discussion environment [12]. The workshop was planned as a 60‐min session, in line with workshop literature [13, 14, 15] and was facilitated by the first author, a registered dietitian and doctoral researcher with training and experience in qualitative research methods, including interviews, Delphi methodology and qualitative thematic analysis. No other members of the research team were present during the workshop. Workshops were guided by a structured discussion guide to support participant engagement and manage group dynamics [13, 14].

A single professional stakeholder workshop was considered appropriate given the aims and scope of this study [13]. As consensus regarding the components of integrated dietetic‐dental care had already been established through a preceding Delphi study [11], the workshop focused on configuring selected full‐consensus agreement statements into an initial conceptual service pathway rather than generating further consensus. Although multiple workshops may support subsequent refinement and implementation planning, the present study aimed to develop an initial conceptual service pathway. Ethical approval was obtained from the University of Plymouth, Faculty of Health Research Ethics and Integrity Committee (Reference: 6495).

Workshops are recognised as suitable approaches for exploring complex healthcare interventions and generating practically‐oriented outputs through collaborative discussions [12, 13, 16]. The use of workshops as a research method was informed by the guidance from O'Cathain et al. [13]. A single workshop was considered appropriate to support configuration of consensus‐derived Delphi findings into a proposed care pathway for paediatric populations attending primary care [13].

### 2.2 Recruitment and Participants

Participants were purposively recruited to capture multidisciplinary perspectives relevant to primary dental care, dietetic practice, service management and integrated care pathway delivery, particularly within the paediatric population [17]. Snowball sampling was additionally used to identify professionals with relevant clinical, operational, educational or research expertise [17]. No financial compensation or incentives were provided and participants were not recruited on the basis of their organisation's plans to implement the proposed conceptual service pathway.

### 2.3 Dietitians

Dietitians were recruited through dissemination of research posters within the British Dietetic Association Paediatric Specialist Group, alongside targeted outreach to professionals identified through publicly available professional roles, networks and relevant publications.

Eligible participants included registered dietitians with experience working with paediatric populations across hospital, community, primary care or private practice settings. Both specialist paediatric dietitians and those in broader clinical roles involving routine paediatric care were included to capture diverse perspectives relevant to integrated paediatric dietetic‐dental care. No minimum years of experience was required. Dietetic support roles were excluded to ensure participation from registered professionals with autonomous clinical decision‐making responsibilities.

### 2.4 Dental Professionals

Dental professionals were recruited from four primary dental care settings in the South West of England where dental students are trained. These settings collectively provide approximately 6260 appointments annually to around 2500 children.

Eligible participants included dental professionals (dentists, dental nurses, dental hygienists and dental therapists) involved in paediatric care delivery and/or research, service coordination, including those holding operational or managerial responsibilities. Professionals with leadership or managerial responsibilities were included due to their insight into organisational processes relevant to pathway development.

### 2.5 Sampling and Recruitment

A target sample of approximately 10 participants was identified, informed by workshop guidance suggesting that smaller groups (3–8 participants) facilitate in‐depth discussion while remaining manageable for collaborative activities [13, 14], while also allowing for potential non‐attendance or participant withdrawal. Eight participants were recruited and attended the workshop, comprising four dietitians and four dental professionals. Interested individuals were provided with an online participant information sheet, consent form and demographic questionnaire prior to workshop participation.

### 2.6 Workshop Development and Data Collection

Workshop activities were informed by the Health Innovation South West, ‘DIY Pathway Mapping Tool’ [15], which provides guidance on mapping care processes and identifying key touchpoints within care pathways. Existing care pathway development frameworks typically assume the presence of an established model of care and a supporting evidence base [18]. As no integrated dietetic‐dental service pathway currently exists within primary dental care, the workshop focused on configuration of selected full‐consensus agreement statements into a conceptual service pathway and identifying organisational and contextual factors [11].

A preceding Delphi study conducted by the authors established 90 statements that achieved full‐consensus agreement regarding the behavioural and organisational components required for integrated care but did not determine how these statements should be operationalised within a coordinated service pathway [11]. Whereas the Delphi study identified *what* components should be included within integrated care, the present workshop determined *how* selected full‐consensus agreement statements should be organised within a conceptual service pathway. Of the 90 statements that achieved full‐consensus agreement, 32 were identified as relevant to the workshop aim of service pathway configuration (Supporting Information S1: Table S1 and Table S6 in reference [11]). These related to referral criteria and processes, professional roles and scope of practice, interprofessional communication, coordination across services and continuity of care. The remaining full‐consensus agreement statements addressed components of the broader integrated care model that were outside the scope of this workshop, including training content, and delivery and procedures relating to anthropometric assessment, and were therefore not incorporated into the workshop prompts.

To support workshop discussion, the first author developed a provisional high‐level pathway organised into four broad stages reflecting the anticipated progression of care (identification, referral, dietetic care and follow‐up). Rather than presenting the 32 selected full‐consensus agreement statements individually, conceptually related statements were synthesised into four concise thematic prompts and aligned with the provisional stages (Supporting Information S1: Table S1). This approach was used to retain the principles represented by the selected statements while providing participants with a manageable basis for pathway‐focused discussion.

This preliminary framework and associated prompts served solely as facilitation tools and were not intended to represent a predefined service pathway. Rather, professional stakeholders used these selected full‐consensus agreement statements as a starting point to configure and refine the sequencing of care activities, patient flow and decision prompts through collaborative workshop activities. Thus, the Delphi study provided the selected full‐consensus agreement statements underpinning the workshop, while their configuration into a coordinated conceptual service pathway was developed through a professional stakeholder workshop, thereby avoiding researcher‐derived assumptions regarding service delivery. Statements that did not achieve full‐consensus agreement in the Delphi study were not considered for service pathway configuration, as the purpose of this phase was to operationalise agreed components rather than incorporate aspects of care that remained contested. The provisional four‐stage framework (Figure 1) functioned as a discussion framework rather than a fixed pathway structure and was iteratively refined throughout the workshop to produce the final conceptual service pathway.

**Figure 1:**
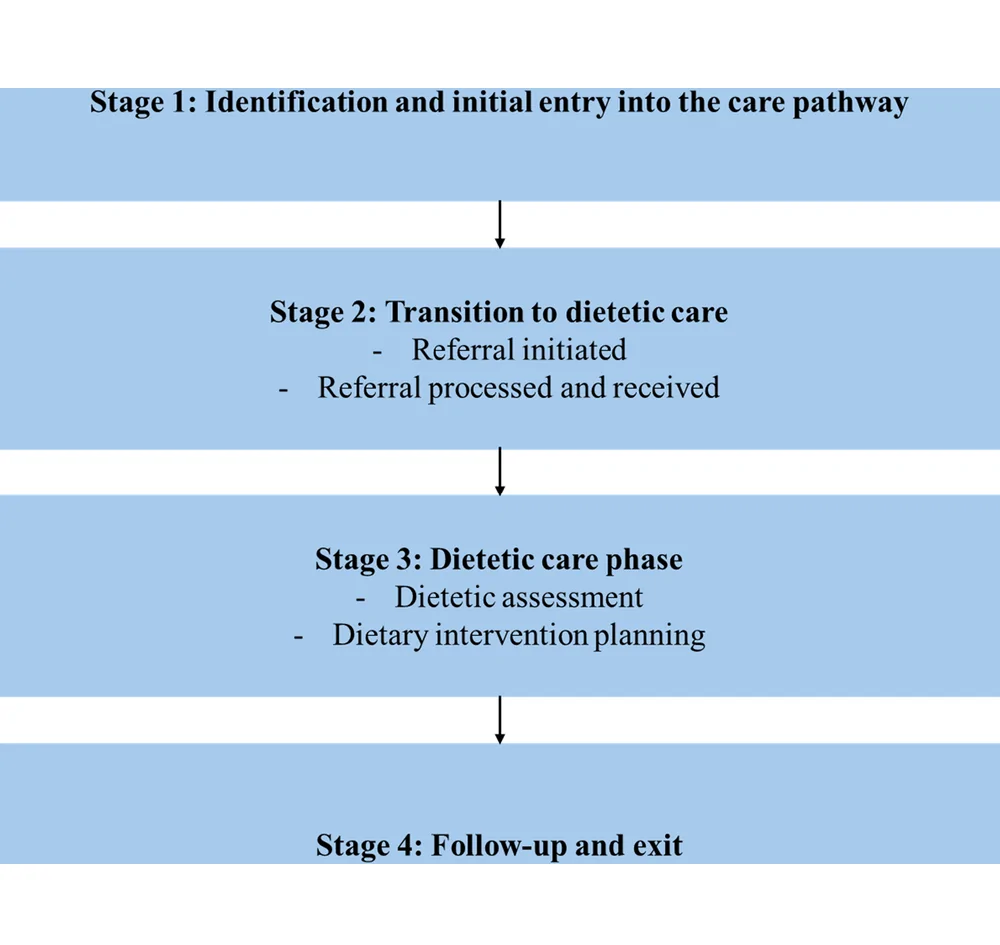
Provisional high‐level pathway developed prior to workshop to support discussion and mapping. The workshop materials presented to participants used the term “care pathway” reflecting the terminology adopted at the time of data collection. Throughout this manuscript, this has been updated to “conceptual service pathway” to more accurately reflect the scope and intended purpose of the proposed model.

Participants were guided to identify potential entry points, decision nodes, convergence points and exit routes within each stage of the provisional high‐level pathway to determine its processes, alongside broader organisational and contextual considerations influencing pathway delivery, as outlined in the workshop facilitation guide (Supporting Information S1: Table S2).

Workshop activities combined individual contributions through written digital sticky‐notes, collaborative discussion and real‐time pathway mapping using a shared digital whiteboard platform (Miro Visual Workspace) (Supporting Information S1: Figure S1) [19]. For each pathway stage, participants first generated individual sticky‐note contributions. These contributions were subsequently mapped in real time by the facilitator into a draft conceptual service pathway, with the organisation and placement guided by participants to support structured group discussion and iterative refinement. The facilitator maintained contemporaneous field notes throughout the workshop.

Data sources included audio‐recorded and verbatim‐transcribed workshop discussions, digital sticky note outputs, pathway mapping artefacts and facilitator notes.

### 2.7 Data Analysis

Data were analysed using the Framework Analysis approach described by Gale et al. [20], enabling systematic integration of multiple qualitative data sources while retaining attention to both the provisional high‐level pathway stages and inductively identified contextual considerations. The workshop audio‐recording was manually transcribed verbatim by the first author and analysed using Microsoft Word (Version 2508). The transcript was checked against the original recording. Familiarisation involved repeated reading of the transcript alongside review of workshop artefacts.

Initial coding was conducted inductively across data sources by the first author to identify factors relevant to care pathway design and delivery, with review from the last author (Supporting Information S1: Table S3). Codes were mapped to either pathway processes or organisational and contextual codes (Supporting Information S1: Table S4). Codes were subsequently reviewed and consolidated into analytical categories (Supporting Information S1: Tables S5 and S6) and mapped onto a working analytical framework, informed deductively by the four provisional high‐level pathway stages: entry into the pathway, transition to dietetic care, dietetic care delivery, and follow‐up and exit (Supporting Information S1: Table S7). Coding remained iterative throughout analysis. Rather than excluding data that did not fit neatly within a single provisional pathway stage, codes were indexed across multiple stages where appropriate, ensuring that all relevant data were retained within the analytical framework. Within each stage, categories related to pathway processes and organisational/contextual influences were developed and applied across all data sources, enabling indexing. Initial coding, framework development and interpretation were reviewed through supervisory discussion to support critical reflection and analytical consistency.

Data were then charted into a framework matrix, whereby evidence from each data source was summarised within the relevant categories and linked to illustrative quotations and workshop outputs. Although all sources informed analysis, they were not considered equally informative. The transcript served as the principal source for understanding participants' reasoning and discussion, while digital sticky notes and pathway‐mapping artefacts supported identification of pathway components. Facilitator field notes provided contextual and reflexive information but were not treated as direct representations of participants' views. The framework matrix facilitated comparison across pathway stages and categories, supporting interpretation of relationships between pathway processes and organisational/contextual influences (Supporting Information S1: Table S8). Interpretation of organisational and contextual influences informing refinement of the conceptual service pathway are presented in Supporting Information S1: Table S9.

Following analysis of the workshop data, a draft service pathway was developed through integration of analysed transcript data and workshop artefacts. The draft conceptual service pathway was subsequently returned to participants for member checking to confirm its clarity, accuracy and completeness. Feedback reviewed through this process was used to refine the pathway where appropriate prior to finalisation but was not treated as a separate dataset for formal qualitative analysis.

Given that the workshop built on selected full‐consensus agreement statements, interpretation focused on understanding how participants configured these within the conceptual service pathway rather than on exploring competing conceptual viewpoints. Where minority perspectives were raised, they were retained within the analytical record and captured as organisational/contextual influences or retained within the service pathway. Framework refinements were reviewed with the research team to support critical reflection and analytical consistency.

### 2.8 Trustworthiness

Several strategies were employed to enhance trustworthiness. A structured facilitation guide informed by Delphi findings supported consistency across workshop discussions. Data synthesis was achieved through the integration and comparison of complementary workshop data sources, including workshop transcripts, digital sticky‐note outputs, pathway mapping artefacts, facilitator notes and member checking feedback. An audit trail documenting pathway development and refinement was maintained through the study. Reflexive notes were maintained throughout data collection and analysis due to the first author's dual role as workshop facilitator and primary analyst [21]. These notes documented observations, assumptions, methodological decisions and reflections on potential influences on data interpretation. The facilitator recognised that her professional background as a dietitian and the focus of the research programme may have predisposed her to view integrated dietetic‐dental care favourably. To minimise the influence of these perspectives, the workshop was structured around discussion prompts derived from the selected full‐consensus agreement statements, participant‐led discussion and collaborative pathway configuration rather than researcher‐directed decision‐making. During analysis, these reflexive notes were revisited to distinguish researcher observations from participant contributions and to support critical reflection on emerging interpretations. Reflexive observations and pathway interpretations were discussed within the research team and reviewed by the final author to challenge assumptions and enhance analytical rigour. Member checking was undertaken following the workshop to enable participants to review the configured conceptual service pathway independently and verify that it accurately reflected the workshop discussions. The workshop involved professionals from different disciplinary and organisational roles, and the potential influence of professional hierarchies was recognised [13]. Participants had no formal professional or supervisory relationship with the first author, who was responsible for participant recruitment and workshop facilitation, although some had participated in earlier phases of the research programme. The remaining authors were not involved in participant recruitment or workshop delivery. Although digital activities aimed to support participation, workshop outputs may still have reflected dominant professional perspectives and existing assumptions regarding service organisation [12].

## 3 Results

### 3.1 Participant Characteristics and Workshop Process

Eight participants took part in the workshop including four dietitians and four dental professionals. Participants represented a range of professional roles including general dental practitioners, paediatric dentist, registered paediatric dietitians and clinic/service leads. Experience ranged from 6 to over 25 years. Full demographic details are provided in Supporting information S1: Table S10.

The workshop comprised participant introductions, an overview of the preceding Delphi study, orientation to the workshop aims and activities and four sequential pathway configuration stages. Introductions and orientation occupied approximately the first 17 min of the session, followed by Stage 1 focus (11 min), Stage 2 (14 min), Stage 3 (17 min) and Stage 4 (9 min). Each pathway stage commenced with approximately 3 min of individual digital sticky‐note generation, followed by collaborative discussion and real‐time pathway mapping within the allocated stage. Following completion of the four stages, participants were invited to contribute assumptions and contextual considerations influencing delivery of the conceptual service pathway, through the shared digital whiteboard. Due to time constraints, consideration of assumptions and contextual factors was included as an optional concluding activity. However, all participants contributed to this stage, and the shared digital whiteboard remained accessible following the workshop to allow any additional reflections. All planned workshop activities were completed within the scheduled session, and no planned workshop stages or activities remained unaddressed. Consequently, no additional workshops were considered necessary.

### 3.2 Overview of Pathway Development

Workshop discussions, sticky‐note outputs (a total of 67 generated across all stages) and pathway mapping findings informed the iterative refinement of the conceptual integrated dietetic‐dental service pathway (Supporting Information S1: Figures S2 and S3). Early mapping activities generated multiple branching referral routes and decision points, which were subsequently refined through data analysis and synthesis. Member checking, resulted in minor refinements to communication loops between dietetic and dental teams, clarification of triage processes and improved representation of contextual considerations within the final conceptual service pathway (Supporting Information S1: Table S11). Figure 2 represents the final conceptual service pathway design.

**Figure 2:**
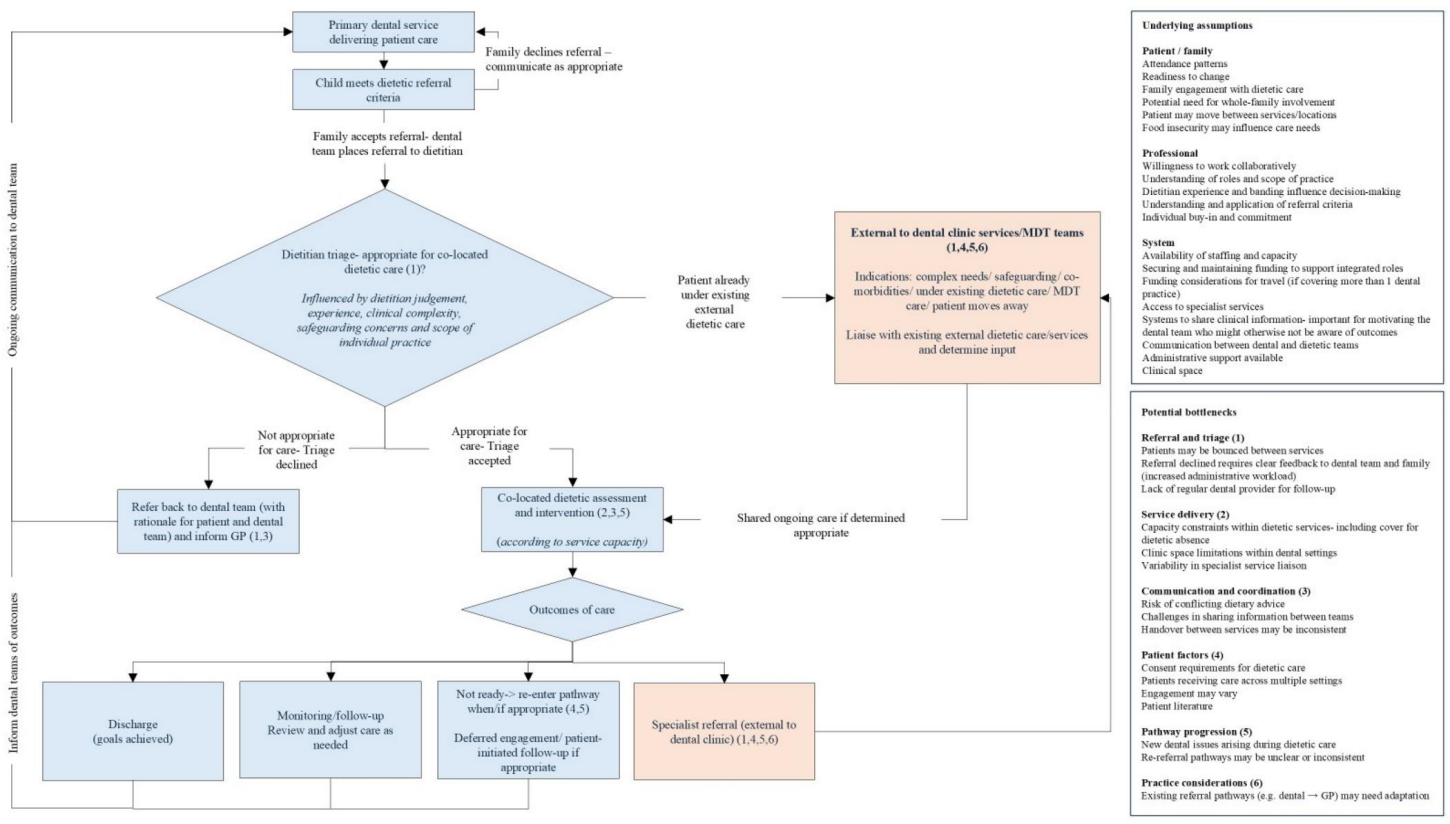
Dietetic‐dental conceptual service pathway. This figure presents the final collaboratively developed integrated dietetic–dental conceptual service pathway, illustrating patient progression from primary dental care through referral, dietetic triage, and subsequent care processes. Dietetic triage determines appropriateness for co‐located dietetic care based on clinical complexity, safeguarding considerations, existing care involvement, and scope of practice. Where co‐located care is not appropriate, patients may be referred back to the dental team or directed to external services. The pathway incorporates bidirectional communication between dental and dietetic teams and links to wider healthcare services. Outcomes include discharge following achievement of treatment goals, ongoing monitoring, referral to specialist services, or deferred engagement with the option for re‐entry. Boxes labelled “Underlying assumptions” and “Potential bottlenecks” summarise contextual and organisational factors identified in the workshop that may influence delivery of the proposed pathway. Numbers within the pathway components indicate where specific assumptions or bottlenecks were raised to particular processes, decision points or transitions of care. Blue boxes represent care delivered within the dental clinic; orange boxes represent care delivered external to the dental clinic. Abbreviations: GP, general practitioner; MDT, multidisciplinary team.

#### 3.2.1 Stage 1: Identification and Initial Entry Into the Care Pathway

Analysis of workshop discussions indicated that pathway entry could not be determined solely by referral criteria. Although oral health‐related referral criteria had been established through the Delphi process, participants consistently identified wider contextual factors influencing progression into dietetic care, including safeguarding concerns, existing dietetic involvement, family preferences and movement across geographical service areas. Consequently, early pathway mapping generated multiple referral routes and decision points to accommodate differing circumstances. Family preferences remained an important consideration through discussions, with the importance of maintaining routes that allow families to decline referral to dietetic care.

#### 3.2.2 Stage 2: Transition to Dietetic Care

As participants considered how this referral complexity could be managed, dietitian‐led triage emerged as a central coordinating mechanism within the pathway. Rather than creating separate referral routes for safeguarding concerns, geographical movement between services or other contextual factors identified during Stage 1, these considerations were viewed as scenarios that could be assessed and managed through triage. Consequently, triage became central to determining referral appropriateness, coordinating onward care and supporting continuity between services.

Early brief conceptual service pathway mapping generated during the workshop highlighted multiple branching entry routes into dietetic care and introduced the concept of triage as a mechanism for determining referral appropriateness (Supporting Information S1: Figure S2). Participant contributions proposed that referrals should be reviewed by a dietitian prior to acceptance, planning and communication with the referring dental professional. Through framework analysis, many of these routes were indicated as considerations influencing referral appropriateness and direction of care rather than requiring distinct pathway stages. Accordingly, they were initially presented as a two‐stage referral and triage process (Supporting Information S1: Figure S3). During member checking, feedback from one participant suggested that the dual triage process could be more clearly represented as a single dietitian‐led triage process and this refinement was incorporated into the final conceptual service pathway (Figure 2).

Participants additionally emphasised the importance of communicating referral outcomes and onward management decisions back to the referring dental professional and family to support continuity and reduce the risk of conflicting dietary advice provided.

So let's say the triage has been done, but the patient doesn't qualify for, you know, treatment under a dietitian. So there will be a letter, I believe, sent back to the person who referred this patient to say that unfortunately the patient doesn't meet the criteria so we won't be able to see them. Can this letter [be] BCC'd to the parents or carers of this patient or to their general medical practitioner so that the patient is somehow aware of the referral has been rejected?

‐ Dental professional

As discussions progressed, practical questions surrounding communication systems and access to patient information became increasingly prominent. While the Delphi process had identified online communication systems as a necessary component of integrated care, and dietitian access to medical records, workshop discussions highlighted uncertainty regarding how information‐sharing would be facilitated across existing organisational systems and geographical regions.

We all use all different notes across all the whole of the UK.

‐ Dietitian

#### 3.2.3 Stage 3: Dietetic Care Phase

Discussions relating to the dietetic care phase focused on the potential complexity of referrals emerging through oral‐health related dietary concerns. While Delphi outputs defined liaison with clinics when children are under existing care or referral when patients are outside the scope of practice, concerns were raised regarding the capacity of existing specialist services to absorb additional referrals generated through earlier identification within dental care.

I think you could uncover something and then not be able to forward it on to the right care.

‐ Dietitian

Participants additionally discussed uncertainty regarding the level of dietetic expertise required within the pathway, including whether delivery would require specialist senior dietitians or lower‐band roles supported by clear escalation processes, dependent on treatment criteria. As discussions progressed, the pathway became increasingly conceptualised as interconnected with wider MDT and multidisciplinary infrastructure and external specialist services rather than what would function as an isolated dietetic‐dental service pathway.

You just have to be really, really clear what the specific treatment criteria is for within these clinics and who you would accept and when you would signpost out and when you'd maybe consider a bit of shared care.

‐ Dietitian

Consequently, the conceptual service pathway was refined to incorporate explicit bi‐directional links with external healthcare services to reflect the expectation that patients may move between co‐located care and wider specialist support depending on complexity, clinical need and scope of the dietitian.

#### 3.2.4 Stage 4: Follow‐Up and Exit

Participants identified multiple potential outcomes following dietetic intervention, including discharge following completion of plan, continued monitoring, escalation to wider services and scenarios where families were not ready to change. During member checking, one participant suggested the incorporation of patient‐initiated follow‐up routes to allow families to re‐access support. This refinement was incorporated into the final pathway.

Workshop outputs additionally reinforced the importance of maintaining communication between dietetic and dental teams following intervention, including the incorporation of feedback loops to support continuity of care and minimise the risk of conflicting advice. Consequently, follow‐up and discharge were increasingly conceptualised as flexible and reversible processes rather than fixed pathway endpoints.

### 3.3 Organisational and Contextual Influences Affecting Care Pathway Delivery

Framework analysis identified several organisational and contextual influences that operated across multiple pathway stages rather than being confined to a single component. During interpretation, these recurring influences were synthesised as either underlying assumptions required for successful pathway delivery or potential bottlenecks that may impede patient progression or care pathway delivery. Figure 2 presents these considerations alongside the final conceptual service pathway and illustrates their relationship to specific pathway processes.

Several assumptions related to patient and family engagement. Progression through the pathway was considered dependent on families' willingness and ability to engage with dietetic care, attend appointments and implement dietary recommendations. Analysis also highlighted that readiness to change, as well as social and family circumstances (e.g., food insecurity), may influence engagement and progression through the pathway. It was also recognised that some families may require ongoing opportunities to re‐engage with support, rather than progressing linearly through care.

Professional assumptions centred on collaborative working and willingness to engage across dietetic and dental teams. The availability of appropriate dietetic expertise was also considered important, particularly given uncertainties regarding the complexity and case‐mix of referrals arising from oral health‐related dietary concerns.

System‐level considerations emerged consistently across multiple stages. Funding, workforce capacity and organisational infrastructure were identified as key important assumptions underpinning pathway delivery. Participants also raised concerns regarding funding requirements beyond the dietetic role, including administrative support, clinic space, transport arrangements and service cover for dietitians.

## 4 Discussion

This study moved beyond identifying acceptable components of integrated dietetic‐dental care to examine what became visible when these components were configured into a conceptual service pathway. The process of pathway configuration revealed forms of coordination work, organisational dependency and contextual uncertainty that were less apparent during the exploratory and consensus phases [7, 11]. These findings suggest that specifying components of integrated care alone does not equate to organisational readiness to deliver them. The conceptual service pathway should therefore be understood as a preliminary model for service design and that future work should define operational processes, including expected triage timelines and urgent escalation protocols and implementation within clinical practice. Although the pathway acknowledges the need for clinical judgement and onward referral in response to clinical complexity, safeguarding concerns and individual scope of practice, the workshop did not seek to establish definitive clinical eligibility or escalation criteria. Such criteria would require further development and local agreement during implementation, informed by clinical governance requirements and the needs of individual services.

### 4.1 Triage Process

Although not predefined within the Delphi findings, triage became necessary as participants worked through how referrals, eligibility decisions and onward communication would operate in practice. Rather than functioning solely as a referral management process, triage evolved beyond a referral management process to function simultaneously as a clinical, administrative and communicative checkpoint linking dietetic and dental care.

The prominence and complexity of triage within the present findings may additionally reflect profession‐specific organisational requirements associated with integrating dietetic services into primary care settings [22, 23]. Although triage processes are common within community dietetic services, literature exploring dietitians integrated within primary care settings suggests that dietetic models cannot necessarily adopt pathways developed for other integrated professions, such as physiotherapists providing first‐contact care, without modification [23]. Nutrition referrals may involve greater uncertainty regarding complexity, referral appropriateness and onward care requirements, often necessitating additional clinical interpretation and allocation processes prior to determining the most appropriate route of care [22, 23]. Oral‐health related dietetic referrals within the present study frequently generated broader considerations regarding feeding complexity, motivation, specialist MDT involvement and onward escalation.

### 4.2 Care Complexity, Dietetic Experience and Case‐Mix

Although full consensus agreement statements concerning referral criteria focused on oral‐health related dietary concerns, workshop discussions highlighted that dietetic assessment initiated within dental settings may uncover broader and more complex presentations including Avoidant/Restrictive Food Intake Disorder (ARFID) or disordered eating related concerns requiring specialist MDT support. It was therefore questioned, as to the extent to which existing specialist services could absorb additional referrals generated through earlier identification within dental care.

Uncertainty regarding referral complexity reflected the novelty of the proposed role and the absence of evidence describing the likely case‐mix emerging through oral‐health related dietetic referrals. While oral disease is associated with wider dietary and nutritional factors, the extent to which broader nutritional or psychosocial complexity may arise remains unclear and may vary according to local oral‐health epidemiology, deprivation and population need [24, 25]. Areas with higher prevalence of childhood oral disease may also experience greater levels of food insecurity, obesity and wider health inequalities, potentially influencing referral volume, workforce requirements and complexity within integrated pathways [26, 27, 28].

Workshop outputs additionally highlighted uncertainty regarding whether the level and type of dietetic expertise that was required within the pathway. One participant also emphasised that management of highly complex paediatric cases often requires input from a broader MDT, rather than relying on specialist expertise operating in isolation. Specialist dietetic services are typically embedded within secondary care MDT structures, raising concerns about the safety and appropriateness of managing more complex cases solely within a co‐located primary care model [29]. Integrated dietetic models within primary care settings have previously been described as “expert generalist” positions, associated with improved patient outcomes and cost savings [22, 30, 31], including within paediatric allergy management [32]. However, the present findings suggest that paediatric referrals related to oral‐health may remain more dependent on specialist MDT support, due to uncertainty around case complexity and patient case‐mix. Consequently, uncertainty extended to whether specialist services have sufficient capacity to absorb more complex referrals. These findings suggest that implementation of an integrated dietetic‐dental service pathway may redistribute, rather than eliminate, complexity within the healthcare system by facilitating earlier identification of broader nutritional, feeding, socioeconomic, psychosocial and motivational concerns at an earlier stage within primary dental care. This has important ethical and organisational implications, as the identification of unmet dietary needs that extend beyond the scope of the integrated dietitian role, in the absence of sufficient capacity within follow‐up services, may create challenges in ensuring appropriate onward support. Effective pathway implementation would therefore require multidisciplinary governance and adequate service capacity to ensure that newly identified needs can be managed safely and appropriately [33].

### 4.3 Material and Organisational Dependencies

The findings additionally highlighted that sustainability of integrated preventive care remained closely tied to wider organisational and material infrastructure within primary dental services. The prominence of organisational and coordination concerns within workshop outputs may partially reflect the professional composition of the participant group, particularly the inclusion of clinicians with operational and service leadership responsibilities. While postulated that investment in preventive dietetic care may reduce restorative treatment burden through earlier intervention and improved dietary behaviours [7], workshop outputs highlighted uncertainty regarding how integrated pathways would be funded and operationally sustained in practice.

One participant reflected that allocating clinic space for dietetic appointments may reduce capacity for income‐generating dental activity. While implications may differ between private and publicly funded dental services, integrated care may require a redistribution of physical and financial resources rather than simply adding preventive care alongside existing provision. Additionally, coordinating access to fragmented health record systems may require further financial investment.

From a dental commissioning perspective, integrated dietetic care may contribute to improved oral health outcomes and support National Health Service (NHS) priorities relating to prevention in children under the age of 5, while potentially reducing restorative treatment burden over time [5, 34, 35, 36, 37]. However, as with wider preventive healthcare models, these potential longer‐term benefits may require substantial initial investment in workforce, infrastructure and clinical capacity before measurable reductions in treatment burden become apparent [36]. Empirical piloting and economic evaluation would therefore be required to determine whether potential longer‐term preventive gains offset shorter‐term organisational and financial pressures, and whether the pathway represents a sustainable use of dental and dietetic resources.

Participants additionally recognised that co‐location may improve the timeliness of dietetic referral, continuity of communication and family engagement through more immediate access to dietary support. From a dietetic commissioning perspective, integrated care may support earlier dietary intervention for children who may not otherwise receive timely dietetic input, whilst simultaneously increasing the identification of more complex nutritional and feeding concerns requiring onward specialist management [22].

Future evaluation should therefore consider outcomes across multiple levels to determine whether the conceptual service pathway is both clinically beneficial and operationally sustainable. At the individual level, potential outcomes could include oral health‐related quality of life, oral health related behaviours, dietary behaviours and clinical oral health measures such as plaque index and decayed missing filled teeth (dmft/DMFT) indices, alongside relevant nutritional indicators where appropriate [38]. At the clinical delivery level, measures such as referral completion, waiting times, uptake and retention, onward referral and staff time could assess pathway feasibility and service efficiency. At the wider service level, evaluation could examine changes in external or secondary care referral demand, caseload mix and complexity and subsequent restorative dental treatment. Economic evaluation could assess the initial investment required for integrated dietetic provision against potential cost offsets associated with avoided or reduced restorative treatment, including consideration of potential return on investment. An implementation framework such as the Consolidated Framework for Implementation Research could serve as a useful theoretical lens to examine organisational, contextual and workforce factors influencing adoption, implementation and sustainability across different service settings [39].

Collectively, the findings suggest delivery of integrated preventive dietetic‐dental care is likely to depend not only on professional agreement regarding referral processes, but also on the availability of coordination infrastructure, workforce capacity, specialist support and organisational systems capable of managing contextual complexity across services.

### 4.4 Limitations and Future Research

Several limitations should be acknowledged when interpreting these findings. The professional groups contributed different forms of contextual knowledge to the workshop. Dietitians were recruited from varied clinical and geographical contexts across the UK, whereas dental professionals were recruited from the South West of England. Consequently, perceptions regarding workforce configuration, communication systems and feasibility were likely shaped by local service contexts and existing interactions with healthcare infrastructure. Several participants also held leadership, educational or service‐management responsibilities, which may have influenced discussions towards operational and contextual considerations. However, these contextual differences also reflect a central finding that configuring integrated preventive care may depend heavily on local organisational systems, referral pathways and available infrastructure. Furthermore, the conceptual service pathway was developed with UK‐based participants and should be interpreted within the context of the UK healthcare system. The geographical concentration of dental professionals in the South West of England is an important limitation to the national transferability of the proposed pathway, as commissioning arrangements, workforce capacity and service infrastructure may differ across regions. Consequently, its transferability to other regions or service configurations within the UK, as well as to other healthcare systems, cannot be assumed and may require adaptation to international and local organisational contexts, workforce models and commissioning arrangements.

Limitations associated with the workshop method should additionally be considered. The workshop represented a one‐off collaborative configuration exercise and therefore captured participant reflections at a single point in time. Its relatively short duration may have increased reliance on the provisional four‐stage framework presented by the facilitator, potentially limiting opportunities to explore alternative models more fully. Although member checking was undertaken, 5 out of 8 participants contributed to this stage, which may have limited opportunities to refine or challenge the final pathway output.

Given the exploratory and professionally focused aims of the workshop, the sample was appropriate for examining operational configuration of the conceptual service pathway. However, broader stakeholder involvement, including caregiver and child perspectives was not integrated within the workshop process. Patient and public involvement was therefore limited. Consequently, the resulting pathway may privilege professional concerns surrounding coordination and organisational processes over experiential aspects of care, including navigation, accessibility and acceptability for families. Future research should incorporate meaningful patient and public involvement, including the perspectives of children and young people and their caregivers, before implementation of the proposed pathway. Such involvement would be important to establish whether the proposed pathway is understandable, acceptable and accessible for families to navigate, and to identify aspects of the pathway that may require adaptation from a family perspective.

Additionally, some workshop participants had contributed to earlier phases of the research programme, whereas others had not. This supported continuity across the sequential stages of pathway development and ensured familiarity with the consensus statements. However, previous participation may also have reinforced existing perspectives and reduced opportunities for independent challenge.

Finally, the conceptual service pathway was intended to support early‐stage professional configuration of a conceptual pathway rather than establish a model of care applicable across all stakeholder groups or healthcare contexts. Substantial uncertainty remains regarding workforce configuration, organisational readiness, referral capacity and system‐level impacts that would require further evaluation during future piloting and implementation work.

## 5 Conclusion

Overall, this study suggests that configuring components represented by the selected full‐consensus agreement statements of integrated dietetic‐dental care into a conceptual service pathway revealed forms of coordination work, organisational dependency and contextual uncertainty that were less apparent during earlier phases of the research. The workshop therefore contributed not only to development of an initial professional configured conceptual service pathway, but also to understanding of the practical negotiation and inter‐service coordination required to deliver integrated preventive dietetic care within primary dental systems. The conceptual service pathway should be understood as an early‐stage service configuration, requiring further adaptation, piloting and evaluation across different organisational and population contexts.

## Author Contributions

All authors conceptualised the manuscript. Lauren Hallewell and Patricia Casas‐Agustench conducted the analysis. Lauren Hallewell drafted the manuscript. All authors critically revised the manuscript. All authors gave their final approval of the final submitted version.

## Funding

The authors have nothing to report.

## Ethics Statement

The study was approved by University of Plymouth Faculty of Health Research Ethics and Integrity Committee (Ref: 6495).

## Conflicts of Interest

The authors declare no conflicts of interest.

## Supporting information


Supporting File

## Data Availability

The data that support the findings of this study are available on request from the corresponding author. The data are not publicly available due to privacy or ethical restrictions.
