# Supplementary material for: Integrating Dietitians Into Primary Dental Care for Paediatric Populations: Developing a Conceptual Service Pathway Through a Professional Stakeholder Workshop
Source: J Hum Nutr Diet. 2026 Oct 9;39(5):e70361. doi: 10.1111/jhn.70361 (PMC13650573; doi:10.1111/jhn.70361)
Supplement: Supplementary file 1 [file JHN-39-0-s001.docx]

**Integrating dietitians into primary dental care for paediatric populations: developing a conceptual service pathway through a professional stakeholder workshop**

**Supporting Information S1:**

Supplementary Table 1. Mapping of the selected full-consensus agreement statements (derived from the Delphi) to the thematic prompts used to facilitate the stakeholder workshop

Supplementary Table 2. Workshop facilitation guide

Supplementary Figure 1. Workshop slide example

Supplementary Table 3. Initial codes generated from workshop data with representative supporting participant evidence

Supplementary Table 4. Grouping of initial codes into pathway processes and organisational/contextual codes

Supplementary Table 5. Refinement of pathway processes into analytical categories

Supplementary Table 6. Refinement of organisational/contextual codes into analytical categories

Supplementary Table 7. Working analytical framework linking analytical categories to the provisional high-level pathway

Supplementary Table 8. Framework analysis matrix showing pathway processes, organisational and contextual influences, supporting evidence and their impact on conceptual service pathway design

Supplementary Table 9. Interpretation of organisational and contextual influences informing refinement of the conceptual service pathway

Supplementary Table 10. Demographic information

Supplementary Figure 2. Conceptual service pathway (Version 1), developed during the workshop

Supplementary Figure 3. Conceptual service pathway (Version 2), shared for member checking

Supplementary Table 11. Member checking Feedback

Note. Workshop materials are presented in their original form as used during data collection and therefore retain the term “care pathway”. Throughout the manuscript and progression of data analysis and synthesis, the terminology was subsequently revised to “conceptual service pathway” to more accurately reflect the scope and intended purpose of the proposed model.

**Supplementary Table 1. Mapping of the selected full-consensus agreement statements (derived from the Delphi) to the thematic prompts used to facilitate the stakeholder workshop**

| Thematic prompt presented at workshop | Selected full-consensus agreement statements informing the prompt |
| --- | --- |
| Referral placed if:   - Oral symptoms affect eating - Repeated dental dietary advice fails or is not implemented - If the family requests support regardless of disease severity - When families express confusion about difficulty implementing dental dietary advice | 9.4 Referral should occur if oral symptoms affect eating  9.5 Referral should happen after repeated dental dietary advice fails or is not implemented  9.7 A child should be referred if the family specifically requests support, regardless of disease severity  9.8 Referral should occur when families express confusion about or difficulty implementing dental dietary advice |
| Facilitating referral and communication:   - Shared digital notes systems accessible to both professions - Integration of communication into existing clinical systems - Structured feedback systems for outcomes and referrals - Used of shared care plans and communication templates - Shared understanding of referral | 16.10 Structured feedback systems for outcomes and referrals  16.11 Use of shared care plans or communication templates  16.12 Clear, localised referral criteria and pathways  16.13 Shared digital notes systems accessible to both professions  16.14 Integration of communication into existing clinical systems  19.1 Shared digital patient records  20.5 Shared understanding of when to refer to dietetics |
| Dietetic care, professional scope and management of complexity:   - Dietetic care involves in-depth dietary assessment and support - Care should be delivered within scope of practice - More complex cases require onward referral to specialist services - Care may involve liaison with other service already involved - Dietetic input may continue alongside other care where complementary support is appropriate | 20.2 Dental professionals should focus on providing brief oral health–focused dietary advice (e.g., sugar frequency, bottle use), while dietitians deliver more in-depth  20.3 There should be a shared understanding across the team about when to escalate from basic dietary advice to a specialist dietetic referral  20.7 Consistent and non-conflicting dietary messages between teams  20.8 All dietary advice provided by the dental team should align with the broader care plan agreed with the dietitian.  20.6 Documenting dietary advice in shared records  20.1 Clear role boundaries  20.11 Clear communication with families about roles  20.4 Referral criteria or flowcharts to guide escalation  11.2 When a child is already under specialist care, the dietitian in the dental clinic should liaise with the existing care team before deciding whether to stay involved  11.3 The dietitian in the dental clinic may remain involved after liaising with the existing team if their input is complementary  11.4 Dietitians in dental clinics should act as bridge between primary care, community and hospital teams, providing light-touch support between specialist reviews  11.5 The dietitian in the dental clinic should provide first-line advice, not work outside their scope of practice and should refer on to specialist services as soon as cases appear more complex  11.8 For children with medical needs affecting diet (e.g., autism spectrum disorder, diabetes, etc.) the dietitian in the dental clinic should support oral health focused advice within their scope  17.1 Access to community and hospital medical records and growth charts  17.3 Connection to specialist dietetic teams (e.g., paediatric, eating disorders)  17.4 The ability to attend local dietetic department meetings  17.5 Dietitians should be linked to dental networks and communication forums for regular interprofessional updates |
| Coordination and continuity of care:   - Care should be coordinated across services - Dietary advice should remain consistent across care providers - Care may continue alongside other services where appropriate - Patient may move between services depending on need - Follow-up should support continuity of care - Clear communication following active involvement, ongoing documentation and between meetings | 17.4 The ability to attend local dietetic department meetings  17.5 Dietitians should be linked to dental networks and communication forums for regular interprofessional updates  18.10 Annual review meetings for evaluation  18.14 Communication between meetings - When significant changes occur in care  18.15 Communication between meetings - ongoing documentation in shared notes  18.16 Communication between meetings - use of flagging systems to alert to dietary issues  17.1 Access to community and hospital medical records and growth charts  17.3 Connection to specialist dietetic teams (e.g., paediatric, eating disorders)  11.2 When a child is already under specialist care, the dietitian in the dental clinic should liaise with the existing care team before deciding whether to stay involved  11.3 The dietitian in the dental clinic may remain involved after liaising with the existing team if their input is complementary  11.4 Dietitians in dental clinics should act as bridge between primary care, community and hospital teams, providing light-touch support between specialist reviews |

The statements were not presented individually to workshop participants. Full-consensus agreement statements relevant to service pathway configuration were grouped according to conceptual similarity and synthesised into concise thematic prompts to facilitate discussion at each stage. Consequently, the relationship between individual statements and workshop outputs is thematic rather than one-to-one.

The numbers in the column “Selected full-consensus agreement statements informing the prompt” refer to the corresponding statements presented in Supplementary Table 6 of Hallewell et al., (2026), “Defining Consensus-Based Components for Integrating Dietitians Into Primary Dental Care for Paediatric Populations: A Delphi Study” ([**https://doi.org/10.1111/jhn.70305).**](https://doi.org/10.1111/jhn.70305)

**Supplementary Table 2. Workshop facilitation guide**

| **Workshop Stage** | **Aim** | **Activities and prompts** | **Outputs generated** |
| --- | --- | --- | --- |
| Introduction | To establish workshop aims, introduce the Delphi process and findings and develop a collaborative discussion environment. | Welcome participants and introduce the workshop purpose. Outline the Delphi findings, clarify the workshop aim to configure care pathway rather than re-negotiate consensus statements, and establish ground rules for respectful discussion and equitable participation. | Shared understanding of workshop purpose, expectations and scope of activities. |
| Stage 1: Identification and initial entry into the care pathway | To identify initial referral routes, decision nodes and patient flow following identification within dental care. | Present Delphi-derived referral criteria and ask participants to consider factors influencing subsequent patient pathways following identification in dental care. Participants then generate individual sticky-note contributions to explore potential decision nodes, convergence points and alternative routes of care. Emerging pathways are collaboratively mapped, discussed and refined in real time. | Visual pathway map, written sticky-note contributions and audio-recorded discussion relating to early care processes. |
| Stage 2: Transition into dietetic care | To explore how patients transition from dental to dietetic care and identify factors influencing care progression. | Present Delphi-derived referral processes and resources. Through sticky-note contributions, participants identify factors influencing transitions into dietetic care, including decision nodes, convergence points, alternative care routes and potential exit points. Emerging pathways are then discussed through facilitated group discussion and subsequently mapped and iteratively refined. | Visual pathway map, written sticky-note contributions and audio-recorded discussion relating to transitions and progression into dietetic care. |
| Stage 3: Dietetic care phase | To determine how dietetic care processes, external service involvement and ongoing management may operate within the pathway. | Present Delphi-derived components relating to dietetic care delivery, liaison with external services and continuation of care. Participants then identify decision nodes, convergence points and exit routes that may influence ongoing patient management. Pathway processes are collaboratively mapped and refined through facilitated discussion. | Visual pathway map, written sticky-note contributions and audio-recorded discussion relating to dietetic care delivery, progression and external service involvement. |
| Stage 4: Follow-up and exit | To identify follow-up processes, discharge routes and potential re-entry into care. | Delphi-informed statements relating to follow-up care, continuity and liaison with other services are presented. Interactive sticky-note activities explore discharge processes, re-entry routes and monitoring considerations. Emerging follow-up and discharge routes are collaboratively mapped, reviewed and refined through group discussion. | Visual pathway map, written sticky-note contributions and audio-recorded discussion relating to follow-up, discharge and re-entry pathways. |
| Contextual factors | To identify barriers, facilitators, assumptions and contextual influences that could affect future delivery of the pathway | Discuss practical factors that could influence the future delivery of the care pathway using sticky-note activities and facilitated discussion. | Written and audio data identifying future delivery considerations. |

**Supplementary Figure 1. Workshop slide example**

This figure shows an example of one of the Miro-based workshop slides used to facilitate collaborative pathway mapping and depicts Stage 4 (“follow-up and exit”) of the process. A provisional four-stage pathway framework (left) was combined with an interactive space for participant-generated sticky notes (right). Full consensus agreement statements from the preceding Delphi study (unpublished data), were presented within a highlighted box (yellow) to support discussion. Contributions were colour-coded by professional group (dietitians: pink; dental professionals: blue) and structured around entry points, decision nodes, convergence points and exit routes.

| **Code** | **Quote/ sticky note**  **Supplementary Table 3. Initial codes generated from workshop data with representative supporting participant evidence.** Representative transcript quotations and workshop artefacts (digital sticky notes) illustrating the development of the initial inductive codes. |
| --- | --- |
| Administrative work | “I feel like you might be creating quite a lot of admin work for yourselves, is my only thought here, because I thought the whole point of having it integrated within the dental service was to try and streamline some of this kind of admin processing. You might be in a fantastic situation, we don't have a lot of admin support at all, so it's always in my mind probably”.- DIET |
| Electronic records | “But if you can get it integrated into your electronic records, then you're all looking at the same thing. So the person who's referred can also see whether it's been accepted. The whole triage process could be on there”.- DIET |
| Access to medical records |  |
| Bouncing between services | “But it's kind of one of them where they might bounce around a little bit and get lost and that's the worry”.- DIET |
| Clinical experience of dietitian |  |
| Being put off attending dentist | “you equally don't want people to not access a dentist or a dietitian because they're scared of a dentist or a dietitian”.- DIET |
| Family motivation | “And some families I think would be really motivated to do some brief intervention at that point because they've had that information and they're generally quite shocked or maybe there's a social worker pressure them to do something and catching them at that point where they've just seen the dentist and the oral health is poor and the growth is poor, that's going to be a really good motivating factor, rather than referring to a local team and waiting three, four months, by which point you've lost that momentum then”.- DIET |
| Staff capacity |  |
| Funding |  |
| Staff motivation |  |
| Conflicting advice |  |
| Other complex social problems |  |
| Involvement of family |  |
| Food insecurity |  |
| Ability to access dental care |  |
| Adapting existing care processes |  |
| Capacity of current services | “Theres lots of emerging conditions such as ARFID, paediatric feeding disorder, all of these issues have never really sort of been identified. previously, or they've certainly worsened since COVID and lots of dietetic departments are feeling very overwhelmed by these types of referrals and quite often they'll need a multidisciplinary feeding team, which often doesn't exist outside big paediatric centres…and we're identifying these in this pathway, we'll be identifying these children through dental care and again, we'll face the same issue of not having, say, an occupational therapist to work with, or a psychologist to be able to manage those kind of disorders. And it's very, very time committing. It uses a lot of capacity to see these patients”.- DIET |
| Existing dietetic involvement |  |
| Safeguarding |  |
| Movement out of care |  |
| Referral acceptance |  |
| Referral criteria |  |
| Triage process |  |
| Feedback to referrer |  |
| Escalation | “It depends what the criteria is on the referral, whether it was related to BMI or what, or it could be that they had additional needs like autism, where it wouldn't be appropriate for us to see them at that stage. It would be more specialist service. So there'd be probably quite a few reasons as to why it may be declined”.- DIET |
| MDT liaison with existing services |  |
| Discharge |  |
| Re-access |  |
| Shared care |  |
| Follow-up |  |

Abbreviations: ARFID, Avoidant/Restrictive Food Intake Disorder; COVID, Coronavirus Disease; BMI, Body Mass Index.

**Supplementary Table 4. Grouping of initial codes into pathway processes and organisational/contextual codes.** Initial codes were grouped into pathway process codes, which informed pathway design and patient flow, and organisational/contextual which captured potential influences on the delivery of the conceptual service pathway.

| **Pathway process codes** | **Organisational/contextual codes** |
| --- | --- |
| Existing dietetic involvement | Administrative work |
| Safeguarding | Electronic records |
| Movement out of care | Access to medical records |
| Referral acceptance | Bouncing between services |
| Referral criteria | Clinical experience of dietitian |
| Triage process | Being put off attending dentist |
| Feedback to referrer | Family motivation |
| Escalation | Staff capacity |
| MDT liaison with existing services | Funding |
| Discharge | Staff motivation |
| Re-access | Conflicting advice |
| Shared care | Other complex social problems |
| Follow-up | Involvement of family |
|  | Food insecurity |
|  | Capacity of current dietetic services |
|  | Ability to access dental care |
|  | Adapting existing care processes |

Abbreviations: MDT, multidisciplinary team.

**Supplementary Table 5. Refinement of pathway processes into analytical categories.** Related codes were synthesised into higher-order analytical categories during framework analysis.

| **Framework categories** | **Codes** |
| --- | --- |
| Referral process | - Referral acceptance - Referral criteria |
| Triage process | - Safeguarding - Movement out of care - Triage process |
| Care coordination | - Existing dietetic involvement - MDT liaison with existing services - Shared care |
| Escalation pathways | - Escalation |
| Post-dietetic care | - Discharge - Re-access - Follow-up |
| Communication processes | - Feedback to referrer |

Abbreviations: MDT, multidisciplinary team.

**Supplementary Table 6. Refinement of organisational/contextual codes into analytical categories.** Related codes were synthesised into higher-order analytical categories during framework analysis.

| **Framework categories** | **Codes** |
| --- | --- |
| Patient/family engagement | - Being put off attending the dentist - Family motivation - Involvement of family - Food insecurity |
| Social complexity | - Other complex social problems |
| Access to care | - Ability to access dental care |
| Collaborative working and buy-in | - Staff motivation - Conflicting advice |
| Professional expertise | - Clinical experience of dietitian |
| Information systems | - Administrative work - Electronic records - Access to medical records |
| Administrative infrastructure | - Administrative work - Electronic records |
| Service capacity | - Staff capacity |
| Funding and resources | - Funding |
| Existing care structures | - Adapting existing care processes |
| External service capacity | - Bouncing between services - Capacity of current dietetic services |

**Supplementary Table 7. Working analytical framework linking analytical categories to the provisional high-level pathway.** Analytical categories were mapped to the provisional high-level four stage framework to support interpretation of pathway processes and organisational/contextual influences.

|  | **Pathway processes categories** | **Organisational and contextual influence categories** |
| --- | --- | --- |
| Stage 1 Identification and initial entry into the care pathway | - Referral process | - Patient/family engagement - Social complexity - Access to dental care - Funding and resources |
| Stage 2 Transition to dietetic care | - Triage process - Care coordination - Communication processes | - Information systems - Administrative infrastructure - Collaborative working and buy-in - Existing care structures - Funding and resources |
| Stage 3 Dietetic care phase | - Care coordination - Escalation pathways - Communication processes | - Professional expertise - Service capacity - Patient/family engagement - External service capacity - Funding and resources |
| Stage 4 Follow-up and exit | - Post-dietetic care - Communication processes | - Patient/family engagement - Access to dental care - Social complexity - Service capacity - Funding and resources |

| **Stage** | **Pathway processes**  **Supplementary Table 8. Framework analysis matrix showing pathway processes, organisational and contextual influences, supporting evidence and their impact conceptual service pathway design**  **(Summary and evidence)** | **Organisational/contextual influences**  **(Evidence)** | **Impact on conceptual service pathway design** |
| --- | --- | --- | --- |
| Stage 1 Identification and initial entry into the care pathway | Participants identified that progression into the pathway was influenced by factors beyond referral eligibility alone. Workshop outputs highlighted the importance of family readiness, consent, safeguarding concerns, existing dietetic involvement and whether children were already receiving care elsewhere. Referral progression was therefore conceptualised as being dependent on both clinical criteria and broader contextual circumstances.  *“But yeah, you've got to keep it [referral criteria] tight, I think, otherwise you could just end up seeing everybody*”.- DIET-3  “*I mean, we got a lot of patients who, you know, in theory, they're agreeing to treatment, but they just don't attend”.-* DENT-4 | **Patient/family engagement**    **Social complexity**    **Access to dental care**    **Funding and resources**   | Initial workshop mapping generated multiple branching referral routes reflecting different circumstances. Through data analysis and pathway refinement, many of these decision points were consolidated within the dietitian-led triage process. Consideration was given to circumstances where families could decline referral, resulting in the incorporation of a feedback loop whereby they do not progress to dietetic triage. |
| Stage 2 Transition to dietetic care | Dietitian-led triage emerged as a central mechanism for assessing referral appropriateness and determining onward management. This included processing information on broader contextual factors, such as family engagement, social complexity and whether the child was already receiving care.  Clear communicating of referral outcomes and triage decisions to dental professionals, families and wider healthcare teams was identified as important to support continuity of care.   | **Information systems**    **Administrative infrastructure**  “*I feel like you might be creating quite a lot of admin work for yourselves, is my only thought here, because I thought the whole point of having it integrated within the dental service was to try and streamline some of this kind of admin processing. You might be in a fantastic situation, we don't have a lot of admin support at all, so it's always in my mind probably”.-* DIET-3  **Collaborative working and stakeholder engagement**    **Existing care structures**    **Funding and resources**   | A dietitian-led triage process was incorporated to assess referral appropriateness, taking account of both clinical and broader contextual factors identified in Stage 1. Communication and feedback mechanisms were integrated to ensure that triage outcomes were communicated to relevant healthcare professionals and families. Patients considered appropriate for co-located dietetic care progressed to the dietetic care phase, while alternative pathways were available where referral was not appropriate. |
| Stage 3 Dietetic care phase | Participants recognised that some children would require referral beyond the integrated service due to complexity or specialist needs and that clear escalation criteria would be necessary. Participants also emphasised the need for communication back to the dental team to support continuity of care.   | **Professional expertise**    **Service capacity**  *“I think you just have to be really, really clear what the specific treatment criteria is for within these clinics and who you would accept and when you would signpost out and when you'd maybe consider a bit of shared care because you equally don't want people to not access a dentist or a dietitian because they're scared of a dentist or a dietitian.”-* DIET-3  **Patient/family engagement**    **External service capacity**  *“Lots of dietetic departments are feeling very overwhelmed by these types of referrals [ARFID/paediatric feeding disorders] and quite often they'll need a multidisciplinary feeding team, which often doesn't exist outside big paediatric centres.*  *and we're identifying these in this pathway, we'll be identifying these children through dental care and again, we'll face the same issue of not having, say, an occupational therapist to work with, or a psychologist to be able to manage those kind of disorders. And it's very, very time committing. It uses a lot of capacity to see these patients, yeah”.-* DIET-2  **Funding and resources**   | Explicit links with external healthcare services were incorporated to accommodate patients requiring specialist input, those already receiving care or those whose needs extend beyond the scope of the dietitian. The pathway also incorporates communication loops back to dental teams to support continuity of care. |
| Stage 4 Follow-up and exit | Participants identified multiple potential outcomes following dietetic intervention including discharge, monitoring, escalation and patient-initiated re-access. Follow-up was increasingly conceptualised as flexible. Clear communication of intervention outcomes, including feedback loops to dental professionals, was considered essential to support continuity of care. | **Patient/family engagement**  **Social complexity**    **Access to dental care**    **Service capacity**  *“I think you could uncover something and then not be able to forward it on to the right care.”- DIET-1*    **Funding and resources**   | Multiple care outcomes were incorporated into the pathway. |

Sticky notes were colour-coded by professional group to support interpretation of contributions: pink represented dietitians and blue represented dental professionals. For facilitator/barrier mapping activities, colour coding reflected the type of influence rather than professional group, with green indicating facilitators/enablers, red indicating barriers/challenges and purple indicating contextual influences.

Some profession-specific sticky notes are presented within the *Organisational/contextual influences* column where they were interpreted as relating to factors influencing pathway delivery, rather than (or in addition to) pathway progression. Quotations were derived from workshop audio recordings.

As shown in the *Organisational/contextual influences* column, several categories were identified across multiple pathway stages. During the interpretive stage of framework analysis, these cross-cutting influences were examined in relation to the proposed pathway and synthesised as either underlying assumptions required for successful pathway delivery or potential bottlenecks that may constrain patient progression or future care delivery. These interpretations informed the assumptions and bottlenecks incorporated into the final care pathway (Figure 2). Abbreviations: ARFID, avoidant/restrictive food intake disorder; GP, general practitioner; PIFU, Patient-initiated follow-up.

**Supplementary Table 9. Interpretation of organisational and contextual influences informing refinement of the conceptual service pathway.** Examination of the framework matrix enabled the identification of organisational and contextual influences operating across pathway stages. These influences were interpreted as either assumptions underpinning successful conceptual service pathway delivery or potential bottlenecks that could impede progression through the pathway. These assumptions and bottlenecks were subsequently incorporated into the final pathway to provider greater transparency.

| **Organisational and contextual framework category** | **Interpretation** | **Representation within Figure 2** |
| --- | --- | --- |
| Patient/family engagement | Assumptions regarding readiness, attendance and engagement. | Underlying assumptions- patient/family  Potential bottlenecks- patient factors (4) |
| Social complexity | Family circumstances may act as barriers to engagement and progression through the pathway. | Underlying assumptions- patient/family |
| Access to dental care | Assumption that the family has regular dental care to attend. | Potential bottlenecks- referral and triage (1) |
| Service capacity | Capacity constraints may affect pathway delivery. | Potential bottleneck- service delivery (2)  Potential bottleneck- pathway progression (5) |
| Funding and resources | There is sufficient and sustainable funding to cover the requirements for integration. | Underlying assumptions- system  Potential bottlenecks- patient factors (4) |
| External service capacity | Referral escalation may be constrained by wider services. | Underlying assumptions- system |
| Professional expertise | Delivery depends on appropriate expertise and service specification. | Underlying assumptions- professional  Potential bottleneck- service delivery (2) |
| Existing care structures | There is an assumption that dental professionals may be required to adapt existing referral processes. | Underlying assumptions- system  Potential bottleneck- practice considerations |
| Collaborative working and buy-in | Assumptions regarding motivation for professionals to work interprofessional. | Underlying assumptions- professional |
| Information systems | Communication relies on access to shared information systems. | Underlying assumptions- system |
| Admin infrastructure | Assumption there is dedicated time and/or sufficient resources to manage administrative workload. | Underlying assumptions- system |
| Care coordination | Assumption that care can be coordinated to prevent conflicting dietary advice. | Underlying assumptions- system  Potential bottleneck- Communication and coordination (3) |
| Communication processes | Information sharing may impede coordination. | Underlying assumptions- system Potential bottleneck- Communication and coordination (3) |

**Supplementary Table 10. Demographic information**

| **Profession** | **n** | **Additional labels** |
| --- | --- | --- |
| Dietitians | 4 | Roles: Registered paediatric dietitian, dietetic service lead/manager |
| Dental professionals | 4 | Roles: General dental practitioner, dental nurse, clinical lead, clinic manager, paediatric dentist, dental educator, lecturer, clinical supervisor |
|  |  |  |
| **Years of professional experience** |  |  |
| 6-10 years | 2 |  |
| 11-15 years | 1 |  |
| 15-20 years | 0 |  |
| 21-25 years | 2 |  |
| 25+ years | 3 |  |
|  |  |  |
| **Region** |  |  |
| South West of England | 4 |  |
| North West of England | 2 |  |
| The West Midlands | 2 |  |
|  |  |  |
| **Work settings** |  |  |
| Primary dental care within a university dental clinic | 4 |  |
| University lecturing | 1 |  |
| Private dietetic practice | 2 |  |
| Industry | 1 |  |
| Community NHS trust | 1 |  |
| Integrated community/acute NHS trust | 1 |  |
|  |  |  |
| **Highest level of education** |  |  |
| General Certificate of Secondary Education  (GCSE; a UK secondary school qualification typically taken at age 16) | 1 |  |
| Undergraduate degree | 2 |  |
| Postgraduate degree | 3 |  |
| Doctorate | 2 |  |

Note: The total number across clinical settings and professional roles may exceed 8, as some participants held multiple professional roles and working across different settings

**Supplementary Figure 2. Conceptual service pathway** **(Version 1), developed during the workshop**

**Supplementary Figure 3. Conceptual service pathway** **(Version 2), shared for member checking**

NB- Dietetic care is delivered by a co-located dietitian within the dental clinic, unless referral to external services is indicated. Blue boxes refer to care being delivered within the dental clinic, while orange boxes refer to care external of the dental clinic.

Numbers in the conceptual service pathway flow, refer to the bottlenecks identified in the box above.

**Supplementary Table 11. Member checking feedback**

|  | Feedback |
| --- | --- |
| Dietitians | Adjustments to conceptual service pathway   - The referral would be received and triaged by a dietitian, which would inform whether the patient is discharged or offered planned treatment. A further stage of triage would not be required unless this has been misinterpreted. If the referral is declined, both the referrer and the general practitioner should be informed. If accepted, no further action would be taken until the initial assessment has been completed and the clinical letter issued. - Any concerns at triage (e.g. safeguarding issues, out of area referrals, or additional needs) would be flagged at this stage. Where administrative support is available, this could be used (or supported by the dietitian) to contact patients and arrange clinic appointments. - If the patient is willing to engage but not ready at that time, they could be added to a patient-initiated follow-up pathway for 6 months, after which they would be discharged if no further contact is made.   Barriers/facilitators/questions   - Clear protocols are needed for managing patient who move out of area or where safeguarding concerns arise. This includes defining how many contact attempts should be made to arrange appointments (e.g. multiple calls versus a single letter or text message), and the timeframe for discharge in the absence of a response (e.g. 14 days). More intensive or extended follow-up may be required in cases involving safeguarding concerns. - Explicit criteria are required to determine when referral to specialist services is appropriate. - A tiered model of care could be considered, with one pathway providing general dietary advice and another offering face to face clinics for patients with more complex needs. - Capacity constrains were identified as a key issue, including: – who will deliver the service, whether care will be provided through a multidisciplinary team involving dietitians and dental professionals. - Clarity is required regarding administrative responsibilities, including booking appointment, following up patients, managing and tracking referrals. - Standardised protocols are needed for the number of appointments offered and management of missed appointments, for example ‘did not attend’ and ‘was not brought’ (DNA/WNB). - Workforce planning should include contingency arrangements for dietetic staff absence (e.g. annual leave, illness). - Securing and sustaining funding for dietetic roles was identified as essential for long-term implementation. - Consideration could be given to establishing multiple dietetic posts across a defined geographical area to provide cross-cover and support shared learning and professional support. - Funding implications should also include travel costs (particularly when multiple dental practices are covered), administrative support and development and provision of patient education materials. |
| Dental professionals | Adjustments to conceptual service pathway   - Within the ‘Outcomes of care’, and specifically ‘Monitoring/ Review and adjust care as needed’, it may be appropriate to include a feedback loop (e.g. an arrow) back to the dental team. This would reflect situations where dietetic recommendations require input or co-operation from the dental professionals (e.g. advice to eat little and often, or the use of high calorie/sugary nutritional supplements). - Under areas identified as ‘difficult to achieve’, it should be acknowledged that implementation of the pathway will require individual commitment and engagement across dental and dietetic services. Effective communication (potentially supported by shared access to patient records) will be important to maintain engagement, particularly where dental professionals may not be aware of patient outcomes.   Barriers/facilitators/questions   - The setting in which dental treatment is delivered is an important consideration. Where care is not provided through Community or Salaried Dental Services, this pathway may be perceived as adding to both administrative and clinical workload without associated remuneration, which could limit engagement from dental teams. |
